# Soil, Leaf and Root Ecological Stoichiometry of *Caragana korshinskii* on the Loess Plateau of China in Relation to Plantation Age

**DOI:** 10.1371/journal.pone.0168890

**Published:** 2017-01-11

**Authors:** Quanchao Zeng, Rattan Lal, Yanan Chen, Shaoshan An

**Affiliations:** 1 College of Natural Resources and Environment, Northwest A&F University, Yangling, P.R. China; 2 Carbon Management and Sequestration Center, The Ohio State University, Columbus, Ohio, United States of America; 3 State Key Laboratory of Soil Erosion and Dryland Farming on the Loess Plateau, Northwest A&F University, Yang ling, Shaanxi, China; Tennessee State University, UNITED STATES

## Abstract

*Caragana korshinskii*, a leguminous shrub, a common specie, is widely planted to prevent soil erosion on the Loess Plateau. The objective of this study was to determine how the plantation ages affected soil, leaf and root nutrients and ecological stoichiometry. The chronosequence ages of *C*. *korshinskii* plantations selected for this study were 10, 20 and 30 years. Soil organic carbon (SOC) and soil total nitrogen (STN) of *C*. *korshinskii* plantations significantly increased with increase in the chronosequence age. However, soil total phosphorous (STP) was not affected by the chronosequence age. The soil C: N ratio decreased and the soil C: P and N: P ratios increased with increasing plantation age. The leaf and root concentrations of C, N, and P increased and the ratios C: N, C: P, and N: P decreased with age increase. Leaf N: P ratios were >20, indicating that P was the main factor limiting the growth of *C*. *korshinskii*. This study also demonstrated that the regeneration of natural grassland (NG) effectively preserved and enhanced soil nutrient contents. Compared with NG, shrub lands (*C*. *korshinskii*) had much lower soil nutrient concentrations, especially for long (>20 years) chronosequence age. Thus, the regeneration of natural grassland is an ecologically beneficial practice for the recovery of degraded soils in this area.

## Introduction

Ecological stoichiometry is the study of the balance of energy and multiple chemical elements in ecological interactions [1]. Ecological stoichiometry connects evolutionary patterns affecting organismal elemental compositions to large-scale consequences and constraints associated with energy and material flows in ecosystems [2]. Ecological stoichiometry, especially for carbon (C), nitrogen (N) and phosphorus (P), plays an important role in analyzing composition, structure and function of a concerned community and ecological system [3]. It has been used in different different systems to explore the limited nutrition for varied plant groups [2, 4–9]. Generally, green leaf N:P ratio can indicate plant nutrition limitation, providing suggestive guide to the practice of vegetation management [3, 9–11]. Leaf N:P ratio (mass ratio) has been suggested to be useful for indicating the shift between N—and P -limitation [12]. Researchers based on fertilization experiments proposed that low leaf N:P ratios (<14) reflect N limitation, that high N:P ratios (>16) likely reflect P limitation[3, 13, 14]. This threshold value has been widely used in different ecosystems.

Vegetation restoration is an effective and beneficial soil conservation practice that can enhance the contents of soil nutrients [15, 16]. The Loess Plateau was the largest eroded land area of China [17]. In 1999, the Chinese government initiated the “Grain for Green” program to address the environmental and economic developmental problems[18]. The program is aimed at converting degraded farmland into forest, shrub, and grassland [19, 20]. Natural grasslands such as the Yunwu Mountain have been protected by establishing shrub land and forest land over large areas [21, 22]. Vegetation restoration has improved soil quality over the last 30 years. Vegetation succession increases small macro-aggregates, bulk soil carbon(C) and aggregate C with an increase in duration of the plantation [23, 24]. Wang et al. (2015)[25] reported that after 15 years of conversion, the natural restoration process must be utilized properly to enhance the accumulation of soil organic carbon (SOC) upon conversion from cropland to grassland. Research studies thus far indicate that vegetation restoration strongly impacts soil quality and the attendant ecosystem services.

*C*. *korshinskii* is the most common shrub which had been established over large areas on the Loess Plateau. Because most studies concerning soil nutrients and aggregate stability in this area, little is known about C:N:P stoichiometry in *Caragana korshinskii* leaf, roots, and soil under different duration age. The duration of the establishment of this shrub varies between 10 and 30 years. Therefore, it is pertinent to assess variations in soil, leaf and root ecological stoichiometry with changes in duration of the plantation. These results will provide well understanding of the practice of planting *Caragana korshinskii* from ecological stoichiometry aspect. In this study, we focused on *C*. *korshinskii* along a successional gradient and a natural grassland with 30 duration years in northwest China. Previous studies had showed that duration age had positive on soil nutrients, especially for the storage of carbon and nitrogen in the grassland [23–25]. Based on the previous studies, we addressed following hypotheses: (1) plantation ages had the positive effects on soil nutrients and ecological stoichiometry; (2) natural restoration might be better method to improve soil nutrients compared with establishing *C*. *korshinskii* for the limited precipitation in drylands.

## Materials and Methods

### Sample sites

*C*. *korshinskii* areas were located in the Shanghuang watershed (106°26′–106°30′ E, 35°59′–36°02′N) in the Loess Plateau, Ningxia province of China, which covered an area of 8.19 hm^2^. This region had a semi-arid climate. The average annual temperature (MAT) was 6.9°C and the average annual precipitation (MAP) was about 419 mm (1982–2002 data) [26]. A severe decline in soil quality and accelerated erosion, serious threats to human wellbeing, were attributed to decline in the vegetative cover because of over-gazing, intensive cultivation and other anthropogenic perturbations. Towards an attempt to address the environmental and economic developmental issues, Shanghuang watershed had been used to conduct restoration projects. Thus, vegetation restoration had been attempted by establishing *C*. *korshinskii*. Thus, the most popular shrub had been planted in the watershed over a wide range of duration. The watershed was highly diverse physiographically and in terrain characteristics spatially (90% of the area is covered by hills, 51% of the land is located at altitude of 1534.3–1822.0 m, and the terrain was closely dissected and sharp-edged with steep slopes) [27]. Restoration of vegetation since circa 1984 had strongly changed the watershed into a demonstration zone for successful land reclamation and comprehensive management. Therefore, the sampling sites for this study ranged in age duration for 10, 20 and 30 years since the establishment of *C*. *korshinskii* plantations after imposing the grazing exclusion.

Another sample site was a natural grassland in the same area, which was a control experiment to compare the effects of vegetation restoration between shrub land and natural grassland. The two sample areas had similar climate (MAP and MAT) and soil types (Entisols, U.S.A. taxonomy). Natural grassland was selected “YunWu Mountain Nature Reserve” where was near Shanghuang watershed. It had a well-protected *Stipa bungeana* population, with an area of 6700 ha, an altitude of 1800–2100 m and an MAT of 6–7°C. The MAP is 455 mm, most of that occurred during the summer (from June to September). In this area, most plants were herb with natural succession, with the constructive species of *Stipa bungeana* and *Stipa grandis*. Other companion species were *Thymus mongolicu*s, *Potentilla bifurca*, *Artemisia vestita*, *Agropyron dasystachys*, *Heteropappus altaicus*, etc [22].

We state that no specific permissions were required for these locations/activities. We conform that the field studies did not involve endangered or protected species.

### Sampling and analyses

We chose three different ages of plots of *C*. *korshinskii* and a natural grassland site without human disturbance as a control which could compare the differences of *C*. *korshinskii* land and grass land duration on soil nutrients and ecological stoichiometry. There were three field plots for every site as repeats. Three subplots (20 × 20 m) were established for each plantation age and NG site, with a total of 36 subplots. In each subplot, we collected three soil and plant samples, respectively. The dominant species and geographic information in each sampling site were showed in Table 1.

**Table 1:** The descriptions of the sample sites.

Revegetation ages/(year)	Height/m	Stand density/(tree/ hm^2^)	Crown area/m^2^	Main types of herb species
10	1.31±0.06	3500±1.53	1.24±0.02	*Stipa bungeana*, *Heteropappus altaicus*, *Thymus mongolicus*, *Artemisia frigida*, *Lespedeza davurica*
20	1.54±0.08	4500±6.08	1.55±0.14	*Stipa bungeana*, *Heteropappus altaicus*, *Artemisia frigida*, *Lespedeza davurica*, *Artemisia scoparia*
30	1.59±0.12	3300±5.29	1.69±0.08	*Stipa bungeana*, *Heteropappus altaicus*, *Lespedeza davurica*, *Artemisia scoparia*
Natural grassland (NG)	-	-	-	*Thymus mongolicus*, *Stipa bungeana*, *Lespedeza davurica*

In natural grassland, we just collected soil samples. Thus, a total of 72 soil samples (under *C.* *korshinskii* and NG), and 54 samples of each of root and leaf for only of *C.* *korshinskii* were collected in this study area in mid-August 2014. Soil and plant samples were collected as previous studies descripted [9, 28]. Briefly, five soil cores (0–20 cm and 20–40 cm depths) were collected from each quadrat using a 3-cm diameter soil auger and mixed to obtain a composite sample. After removing stones, roots and small animals, soil samples were air-dried and sieved through a 0.15 mm sieve for the analysis of soil organic carbon, total nitrogen and total phosphorus. Plant samples included green leaf and root samples. In each subplot, we collected green leaf and root from 5 well-grown *C*. *korshinskii*. All the roots were fine roots with the diameter of < 0.5 mm. Leaf and root were watering with distilled water and dried at 65°C for about 72 hours until reached a consistent weight. After over-dried, leaf and root samples grounded using a ball mill and sieved through a 0.15 mm sieve.

Concentrations of total nitrogen (TN) of the plant tissues and of soil total nitrogen (STN) were determined colorimetrically using the Kjeldahl acid-digestion method (KDY-9830) after extraction with 0.02 mol/L sulfuric acid [29]. Total plant P (TP) was measured using colorimetric analysis (UV 2800) after digestion with H_2_SO_4_ and H_2_O_2_ [30]. Soil total P (STP) was measured using a spectrophotometer after wet digestion with H_2_SO_4_ and HClO_4_ followed by colorimetric analysis (UV 2800) [31]. The organic C concentration in soils and plants was measured using a modified Mebius method [32]. More specifically, 0.5 g of soil samples (or 0.02 g of plant samples) were digested with 5 ml of 1 mol/L K_2_Cr_2_O_7_ and 5 ml of concentrated H_2_SO_4_ at 180°C for 5 min, followed by titration with standardized FeSO_4_ (0.2 mol/l).

### Data analysis

We used one-way analysis of variance (ANOVA) to analyze the effects of duration age on nutrients and stoichiometric characteristics of the soil, root, leaf. Linear regression analysis was used to test the relationships between the soil and plant stoichiometric characteristics. All the significant differences were showed at the level of 0.05. SPSS 20.0 software (SPSS, Inc., Chicago, IL, USA) was used for the statistical analysis. Soil and plants nutrients concentrations expressed as g/kg on a dried weight basis and all the C:N:P ratios were mass ratios. Figures were conducted by Origin 9.0.

## Results

### Soil nutrients and soil C:N:P characteristics

**Fig 1:**
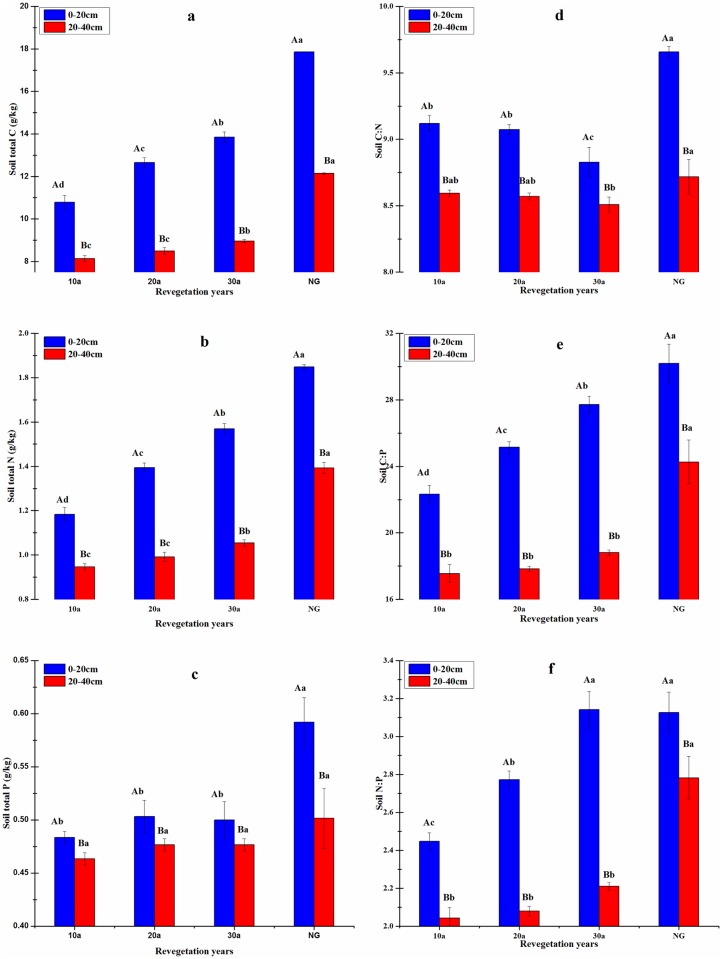
Stoichiometric characteristics of soil C, N and P as affected by soil depth and plantation age. Values are the means ± SE of three plots. Values designated by different lowercase letters were significantly different among different duration ages, and different capital letters indicated significant difference soil layers, respectively (P < 0.05).

The plantation age had significant effects on the soil organic carbon (SOC) and soil total N (STN) concentrations (Fig 1). Concentrations of SOC (g/kg) for 10, 20 and 30 yr duration were 10.79, 12.66 and 13.85 in the 0-20-cm soil layer compared with 8.13, 8.49, and 8.97 in 20-40-cm layer, respectively. Concentrations of STN increased with increase in the plantation age, and the highest value was measured at 30 yr. Concentration of STN (g/kg) for 30 yr was, 1.57 and 1.05 in 0–20 cm and 20–40 cm-soil layer, respectively. In comparison, concentration of STP had a narrow range (0.46–0.50 g/kg) in 0-20-cm and 20-40-cm soil layers. While concentrations of SOC and STN changed significantly (*P*<0.05) with increase in the plantation age (from 10 yr to 30yr), that of STP had no significant effect. However, concentrations of SOC and STN under NG were approximately 2-fold higher than those measured under *C*. *korshinskii* plantations. Similarly, concentration of STP under NG was much higher than that measured under three *C*. *korshinskii* sites of 10 yr, 20 yr, and 30yr.

The soil ecological C:N:P stoichiometry also varied with the plantation age (Fig 1). The soil C:N ratios for plantation age of 10, 20 and 30 yr under *C*. *korshinskii* were 9.12, 9.07, and 8.80 in the 0-20-cm soil layer, respectively. However, the C:N ratio was rather narrow (8.51–8.59) in 20-40-cm layer. Overall, along the plantation age significantly impact the soil C:N ratio. In fact, the trend of the soil C:P ratio was also similar to that for soil N:P ratio (Fig 1). The soil N:P ratio significantly increased with increase in the plantation age for both soil depths (Fig 1). The C:N:P ratio also increased with increase in the plantation age (Table 2).

**Table 2:** Stoichiometric characteristics of plant and soil C:N:P as affected by plantation age and soil depth.

Variable		C:N:P			
		10-year-old	20-year-old	30-year-old	NG
Leaf		291:24:1	258:23:1	247:22:1	-
Root		659:31:1	530:27:1	434:25:1	-
Soil depth	0–20 cm	22:2:1	25:3:1	28:3:1	30:4:1
	20–40 cm	18:2:1	18:2:1	19:2:1	24:3:1

### Leaf and root C, N and P contents and C:N:P characteristics

**Fig 2:**
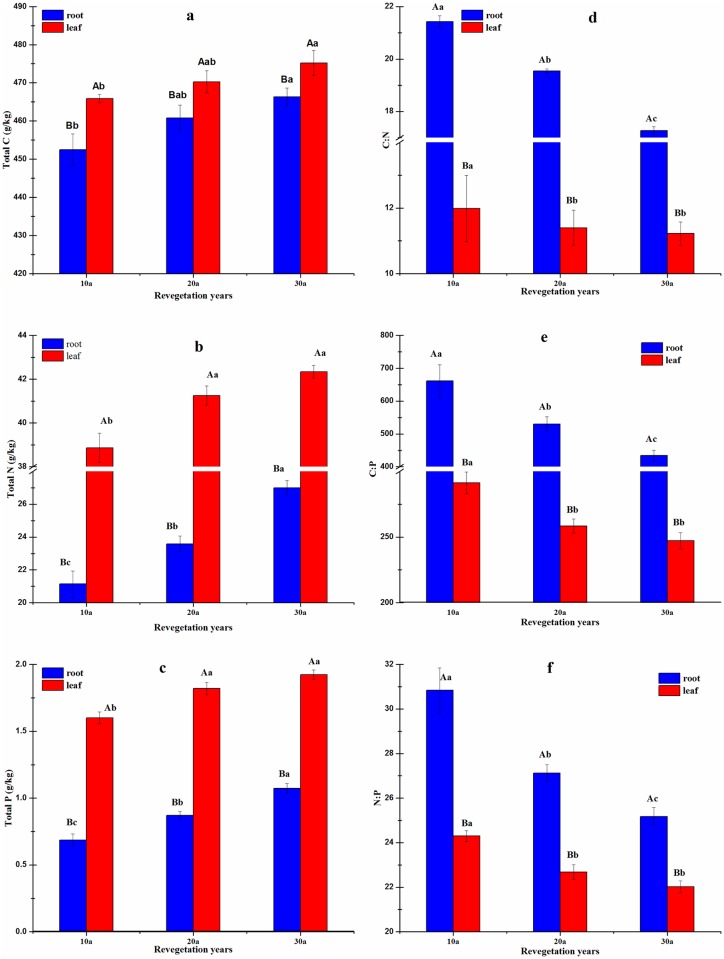
Stoichiometric characteristics of leaf and root C, N and P as affected by plantation age. Values are the means ± SE of three plots. For each plant issue, means with different letters are significantly different based on ANOVA and Scheffe’s test (*P*<0.05). Note: Values designated by different lowercase letters were significantly different among different duration ages, and different capital letters indicated significant difference between leaf and root, respectively (*P* < 0.05).

The plantation age had a significant effect on leaf total C, total N and total P concentrations. The highest concentrations (g/kg) of total C (475.2), total N (42.3) and total P (1.9) were observed under the 30-year-old and the lowest under 10-year-old plantation of *C*. *korshinskii* (Fig 2). Concentration (g/kg) of nutrients in roots ranged from 452.3 to 466.3 for total C, 21.2 to 27.0 for total N, and 1.6 to 1.9 for total P.

Increase in the plantation age decreased the ratio of nutrients in the leaves from 12.0 to 11.2 for C:N, 291.3 to 247.1 for C:P, and 24.3 to 22.0 for N:P. Trends in the ratio of nutrients in the roots with regards to the planation age for C:N, C:P and N:P exhibited trends similar to those of the leaves. The plantation age had a significant effect on the leaf and root ecological stoichiometry (*P*<0.05).

The highest leaf C:N:P ratios were observed in the 30-year-old and the lowest in the 10-year-old plantation (Table 2). Trends of the root C:N:P ratios were similar to those of the leaves (Table 2). In general, the plant C:N:P ratios increased with increase in the plantation age.

### Relationships between soil versus leaf and root stoichiometry

**Fig 3:**
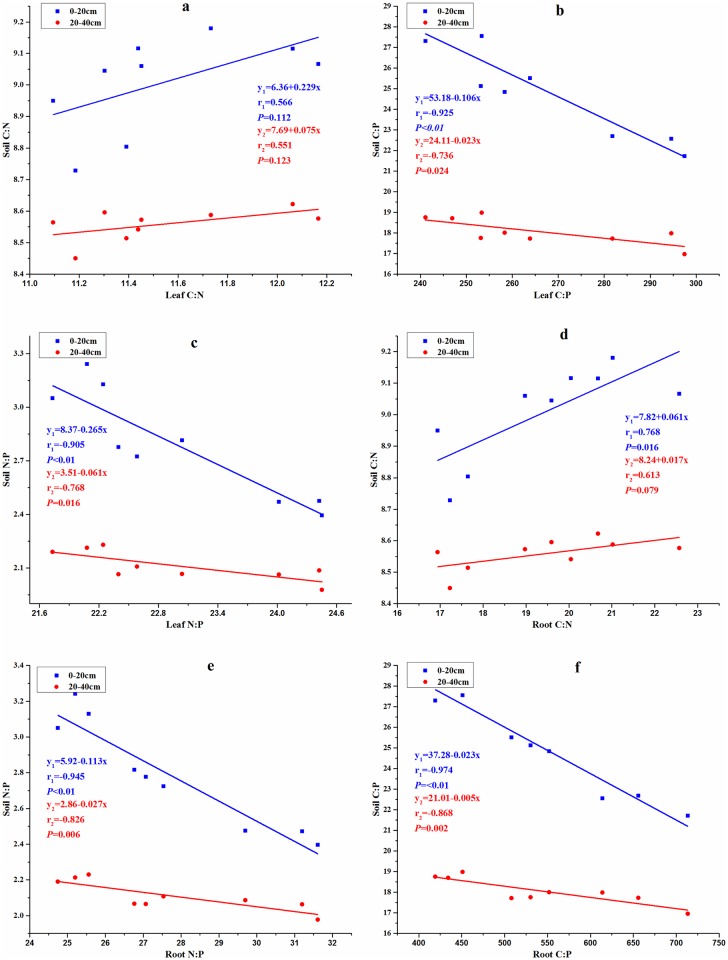
Relationships between soil, leaf and root stoichiometric characteristics of *C.korshinskii*.

The leaf C:P ratio decreased linearly with the soil C:P ratio (r = -0.925, *P*<0.01 for the 0-20-cm; r = -0.736, *P* = 0.024 for 20-40-cm layer) (Fig 3b). The N:P ratio in soil under *C*. *korshinskii* varied linearly with the leaf N:P ratio (r = -0.905, *P*<0.01 for the 0-20-cm; r = -0.768, *P* = 0.016 for 20-40-cm layer) (Fig 3c). In the 0-20-cm layer, the soil C:N ratio increased linearly with the root C:N ratios (r = 0.768, *P* = 0.016) (Fig 3d). The soil N:P ratios decreased linearly with the root N:P ratios (r = -0.945, *P*<0.01 for 0-20-cm; r = -0.826, *P* = 0.006 for 20-40-cm layer) (Fig 3e). The soil C:P ratios decreased linearly with the root C:P ratios (r = -0.974, *P*<0.01 for 0-20-cm; r = -0.868, *P* = 0.002 for 20-40-cm layer) (Fig 3f).

## Discussion

### The effects of plantation ages of *C. korshinskii*

Vegetation restoration was one of the most effective method to solve the soil erosion problem, especially on the Loess Plateau of China [33, 34]. Shrubs (i.e. *C.korshinskii*) were one of the most common vegetation types in the Shanghuang watershed and had been used to improve the soil quality, including soil aggregates and nutrient availability. The data from the present study showed that vegetation age had positive effects on soil nutrients, and these results were in accord with those reported for the Shapotou area [35], Yaoledianzi village in northeast China [36], Wulanaodu area in northeast China [37], and Yanchi country in Ningxia province, northwest China [38]. Revegetation from abandoned farmland significantly influenced soil total C and total N, resulting in a C:N ratio of about 10 after 30 years of abandonment [39]. The observations in this study showed that the C:N ratio of soil under *C*. *korshinskii* ranged from 8.80 to 9.11. In the first 20 years, soil C:N ratio was nearly constant, and significant higher than the soil in the duration of 30 years. Soil total C concentration was significantly correlated with the soil total N, indicating similar trends for soil C and N storage. This coupling relation led to a narrow range for soil C:N ratio.

The N: P ratio in leaf was an indicator of N or P limitation in terrestrial ecosystems [13]. The relationships between leaf N: P ratios and plant properties have also been used to indicate their responses to environmental change or human disturbance [40]. In this study, the N:P ratios decreased with increase in plant age, and all the ratios were higher than 20. Review of previous studies [3, 14], showed that *C*. *korshinskii* was limited by P nutrition, and the severity of limitation decreases with the increase in age of the shrub, implying that older *C*. *korshinskii* plants need a much lower available P requirement for growth. Therefore, soil available P content would increase strongly with increasing *C*. *korshinskii* age, and which had positive consequences for soil quality. However, it was interesting that soil total P concentration was not decreased with age increase. Because soil parent materials were the main resources of total P content[41]. Thus *C*. *korshinskii* soil total P had no significant changes with age increase.

### The difference between *C. korshinskii* and natural grassland

Land use was another factor affecting soil nutrients. Grasslands played an important role in the global C and N cycles. The data showed that soil total C and total N were higher in the older grasslands (30 years) and the values were much higher than in a soil with similar aged *C*. *korshinskii* plots. Thus, vegetation type had a strong effect on the soil C and N storage. Li et al. (2013) [42] reported that C input was greater than C output in an enclosed grasslands. Soil N fixation by *C*. *korshinskii* and subsequent N released by litter decomposition could increase soil N content [36]. Carbon fixation via photosynthesis and the subsequent transfer of C to the soil via leaf, litter and root turnover contributes to soil C accumulation [43]. A larger aboveground biomass indicated that more litters returned to the soil and the roots exudates would contain more nutrients.

At the same age, soil under natural grassland had the highest total C, total N and total P, suggesting that, compared with natural grassland, *C*. *korshinskii* was not the most suitable specie for restoration of eroded lands in this region. Firstly, *C*. *korshinskii* required more amount of water and nutrients to maintain its growth than grasses for the higher living biomass. Secondly, given that plant growth was main limited by water in the dryland [44], the annual precipitation was limited in this area. Finally, soils of the studied areas were deficit in nutrients before planting *C*. *korshinskii* because of over grazing, which also limited the growth of *C*. *korshinskii*. Therefore, natural restoration may be the best approach to restore degraded soils to attain the desired outcome albeit taking a long time. While establishing *C*. *korshinskii* improved soil quality over a short time period, it had negative effects on the soil on the long-term basis. Consequently, planting *C*. *korshinskii* was not the best choice to improve soil quality for Shanghuang watershed in the long run.

## Conclusion

*C*. *korshinskii*, a leguminous shrub, is a dominant native plant species that is widely planted to prevent soil erosion on the Loess Plateau. The results showed that *C*. *korshinskii* growth altered concentration of nutrients in soil, leave and root. The leaf N:P ratios were higher than 20 regardless of the age of the shrub. Therefore, the growth of *C*. *korshinskii* was limited by P availability. Compared with natural grassland, planting *Caragana korshinskii* scrubland may not be a good choice for vegetation restoration as its soil fertility was lower than the natural grasslands. Accumulation of soil nutrients under *C*. *korshinskii* is a slow process and takes a long time.

## Supporting Information

S1 File Supporting information about data. (XLSX)
